# Trends in and drivers of healthcare expenditure in the English NHS: a retrospective analysis

**DOI:** 10.1186/s13561-020-00278-9

**Published:** 2020-06-30

**Authors:** Idaira Rodriguez Santana, María José Aragón, Nigel Rice, Anne Rosemary Mason

**Affiliations:** 1 HCD Economics, The Innovation Centre, Keckwick Ln, Daresbury, Warrington, WA4 4FS UK; 2 Centre for Health Economics, Alcuin A Block, University of York, York, YO10 5DD UK

**Keywords:** Healthcare expenditure, Activity, Cost, Drivers, Demographic pressures, Technology

## Abstract

**Background:**

In England, rises in healthcare expenditure consistently outpace growth in both GDP and total public expenditure. To ensure the National Health Service (NHS) remains financially sustainable, relevant data on healthcare expenditure are needed to inform decisions about which services should be delivered, by whom and in which settings.

**Methods:**

We analyse routine data on NHS expenditure in England over 9 years (2008/09 to 2016/17). To quantify the relative contribution of the different care settings to overall healthcare expenditure, we analyse trends in 14 healthcare settings under three broad categories: Hospital Based Care (HBC), Diagnostics and Therapeutics (D&T) and Community Care (CC). We exclude primary care and community mental health services settings due to a lack of consistent data. We employ a set of indices to aggregate diverse outputs and to disentangle growth in healthcare expenditure that is driven by activity from that due to cost pressures. We identify potential drivers of the observed trends from published studies.

**Results:**

Over the 9-year study period, combined NHS expenditure on HBC, D&T and CC rose by 50.2%. Expenditure on HBC rose by 54.1%, corresponding to increases in both activity (29.2%) and cost (15.7%). Rises in expenditure in inpatient (38.5%), outpatient (57.2%), and A&E (59.5%) settings were driven predominately by higher activity. Emergency admissions rose for both short-stay (45.6%) and long-stay cases (26.2%). There was a switch away from inpatient elective care (which fell by 5.1%) and towards day case care (34.8% rise), likely reflecting financial incentives for same-day discharges. Growth in expenditure on D&T (155.2%) was driven by rises in the volume of high cost drugs (270.5%) and chemotherapy (110.2%). Community prescribing grew by 45.2%, with costs falling by 24.4%. Evidence on the relationship between new technologies and healthcare expenditure is mixed, but the fall in drug costs could reflect low generic prices, and the use of health technology assessment or commercial arrangements to inform pricing of new medicines.

**Conclusions:**

Aggregate trends in HCE mask enormous variation across healthcare settings. Understanding variation in activity and cost across settings is an important initial step towards ensuring the long-term sustainability of the NHS.

## Background

Since the NHS was established in 1948, healthcare expenditure (HCE) has risen faster than both GDP and total public expenditure [1], a trend that is echoed in most OECD countries [2]. Between 2008 and 2018, government expenditure on healthcare in England rose 25% in real terms, substantially more than the 13% real terms growth of the economy (GDP), and faster than every other category of government expenditure [3]. Rises in HCE are expected to continue in the medium to long-term even in the most conservative cost containment scenarios [2].

Tackling the drivers of HCE is an enduring policy concern. Known drivers of overall growth in HCE include behaviours and lifestyle factors such as smoking, diet or physical activity [4], wealth and income effects [5] and prices [6]. There is evidence that demographic factors such as population ageing [7] are associated with rises in HCE. Increases in the prevalence of multimorbidity is another well-known predictor and studies suggest that comorbidities may be ‘super-additive’ meaning that the total cost of treating comorbid conditions is greater than the sum of the independent treatment costs of the underlying disease conditions [8]. More recently, macro-level studies of US expenditure have identified strong positive relationships between HCE and technological progress [5, 9], although the impact of new technology appears to vary across the distribution of expenditure [10].

Year-on-year real term rises in HCE, such as those observed within the English NHS, are considered to be one of the greatest challenges to its long-term fiscal sustainability [11]. To ensure the NHS remains financially viable, there is a need to understand how HCE may change in the future. This requires an oversight of historical trends in activity and cost across the whole system, and an appreciation of how these vary by healthcare setting and why. For example, a disaggregated analysis may reveal settings where costs are rising but activity is static, and this may be due to inefficiencies and/or waste. According to the OECD, one-fifth of health spending is wasteful; examples include missed appointments, avoidable admissions, duplication of services, delayed discharges and unnecessary expenditure on pharmaceuticals or procedures of limited clinical value [12].

A simple comparison of trends across healthcare settings can identify “pressure points” and help to guide an exploration of potential drivers leading to improved performance. In addition, understanding how trends in expenditure, activity and cost vary across settings can inform spending reallocations within existing budgets, and improve workforce and budget planning.

However, few studies of drivers of HCE have investigated how factors vary by care setting [3, 10, 13]. In addition, analyses of HCE trends are commonplace, but rarely disaggregate HCE growth into its constituent parts: activity and costs. The purpose of this study is to address those gaps in the evidence base. Our analyses provide an overview of the trends in expenditure and their breakdown in terms of cost and activity growth in three broad categories of care in the English NHS between 2008/09 and 2016/17. These categories together account for over 80% of total NHS spend. For each of the three categories, we also analyse trends in healthcare settings, and identify potential drivers for the observed trends drawing on evidence from the published literature.

## Methods

To quantify the relative contribution of different settings to overall HCE, we analyse trends in expenditure, activity and costs for 14 healthcare settings of the English NHS. The settings are grouped into three broad categories: Hospital Based Care (HBC), Diagnostics and Therapeutics (D&T) and Community Care (CC). The study period covers the financial years 2008/09 to 2016/17. Potential drivers for the observed trends are identified from evidence in the published literature.

Table 1 shows which settings are included in each of the three categories, and the type of activity captured by each setting.

**Table 1:** Rates of growth in English NHS expenditure, activity and cost by healthcare setting

Category	Setting	Type of Activity	Total Growth 2008/09–2016/17			Mean year on year growth 2008/09–2016/17		
			Expenditure	Activity	Cost	Expenditure	Activity	Cost
Hospital Based Care (HBC)	Inpatient Care	FCE and Excess bed days	38.6%	19.5%	16.0%	4.2%	2.3%	1.9%
	Outpatient	Attendances and procedures	57.2%	43.7%	9.4%	5.8%	4.7%	1.1%
	Accident & Emergency	Attendances, investigations, treatments	59.5%	30.2%	22.5%	6.0%	3.4%	2.6%
	Specialist Services	Activity	34.8%	21.7%	10.8%	3.8%	2.5%	1.3%
	*HBC weighted average growth*		54.1%	29.2%	15.7%	5.6%	3.3%	1.8%
Diagnostics and Therapeutic (D&T)	Chemotherapy	Treatment, procurement	113.1%	110.2%	1.4%	10.0%	9.9%	0.4%
	Radiotherapy	Treatment, preparation	42.9%	72.1%	−17.0%	4.6%	7.3%	−2.2%
	High Cost Drugs	Drug types	230.7%	270.5%	−10.7%	16.7%	18.0%	−1.2%
	Radiology	Examinations	34.1%	39.8%	−4.1%	3.8%	4.3%	−0.5%
	Diagnostic Tests	Tests	47.3%	59.0%	−7.4%	5.1%	6.2%	−0.8%
	Renal Dialysis	Sessions	16.1%	−1.0%	17.3%	1.9%	−0.1%	2.0%
	*D&T weighted average growth*		155.2%	191.1%	−7.0%	12.5%	14.4%	−0.9%
Community Care (CC)	Community Prescribing	Prescriptions	9.8%	45.2%	−24.4%	1.2%	4.8%	−3.4%
	Community Services	Activity	35.0%	18.7%	13.8%	4.0%	2.4%	1.6%
	Optometry & Dentistry	No. eye tests and dental procedures	23.7%	7.2%	15.3%	2.7%	0.9%	1.8%
	Rehabilitation	Activity	10.4%	−2.3%	13.1%	1.5%	−0.1%	1.6%
	*CC weighted average growth*		19.2%	34.7%	−7.1%	2.3%	3.8%	−0.9%
	Total: all settings		50.2%	40.3%	7.1%	5.2%	4.3%	0.9%

*FCE* Finished Consultant Episode.

Two important settings, primary care and community mental healthcare, have been excluded from the analysis. This is due to a lack of historical official estimates of activity and cost for primary care and a lack of data for community mental health before 2011/12.

### Data

For 12 of the 14 settings, activity and cost data come from the National Schedule of Reference Costs [14]. NHS providers are required to report these administrative data every year in accordance with national costing guidance. The cost of High Cost Drugs is included in the National Schedule of Reference Costs. Data on community prescribing comes from the Prescription Cost Analysis (PCA) dataset [15],^1^ which provides details of the number of items and the net ingredient cost of prescriptions dispensed in the community. Data on activities and costs of dentistry [16] and optometry [17] are provided by NHS Digital.

### Measuring trends in activity and cost

In order to disentangle the extent to which changes in HCE are driven by changes in activity and/or changes in unit cost we employ a set of indices. These are measures of change that allow the aggregation of diverse output items (such as Finished Consultant Episodes (FCEs), attendances, tests, prescriptions, etc.) in a single index and are useful for facilitating comparisons across categories and settings of healthcare. These indices are routinely used in healthcare productivity analyses to measure the rate of growth of output [18, 19].

The Laspeyres Activity index is shown in Eq. 1. Cost is held constant to quantify the change in activity: the denominator is the product of each type of activity at time 0 and its associated cost at time 0; the numerator is the product of activity at time t and its cost at time 0. The Paasche Price index (Eq. 2), works in a similar way, but activity is held constant to quantify the change in cost. The index for Total Expenditure incorporates both cost and activity changes (Eq. 3).

*Equation**1**(i) Laspeyres Activity Index*

1 $$ {X}_{\left(0,t\right)}^L=\frac{\sum \limits_{j=1}^J{x}_{jt}{c}_{j0}}{\sum \limits_{j=1}^J{x}_{j0}{c}_{j0}} $$

*Equation**2**(ii) Paasche Cost Index*

2 $$ {C}_{\left(0,t\right)}^P=\frac{\sum \limits_{j=1}^J{x}_{jt}{c}_{jt}}{\sum \limits_{j=1}^J{x}_{jt}{c}_{j0}} $$

*Equation**3**(iii) Total Expenditure Growth*

3 $$ {E}_{\left(0,t\right)}={C}_{\left(0,t\right)}^P\ast {X}_{\left(0,t\right)}^L=\frac{\sum \limits_{j=1}^J{x}_{jt}{c}_{jt}}{\sum \limits_{j=1}^J{x}_{j0}{c}_{j0}} $$

In all three equations, *x*_*j*_ is the number of units of activity, i.e. FCEs, attendances, or treatments of type *j*, where *j* = 1, …, *J*; *c*_*j*_ is the unit cost of output *j*; and *t* is time with *t* = 0 indicating the first period of the time series. The formulae are shown for a two-period index. To measure growth over a longer period of time, we use a chain index. In a chain index, the computation of the growth rates is performed over successive periods, then the product of these growth rates produces a chain series that uses the first period as reference (i.e. base year). Equation (4) shows the chain for the Laysperes activity index.

*Equation**4**Chain index for Laspeyres Activity*

4 $$ {X}_{\left(0,T\right)}^L={X}_{\left(0,t\right)}^L\times {X}_{\left(t,t+1\right)}^L\times \dots \times {X}_{\left(\mathrm{T}-1,T\right)}^L $$

We calculate these three indices for each of the three broad categories of care HBC, D&T and CC, and also for the 14 subcategories (settings). We then plot growth rates using 2008/09 as the base year (i.e. 2008/09 indices are set equal to 100). Next, we identify relevant setting-specific evidence, drawn primarily from a previous review [3], to identify potential drivers of the observed trends. All analyses were conducted using SAS Enterprise Guide 7.1.

### Identifying drivers of trends in activity and cost

To identify potential drivers for the observed trends we drew on a previous systematic review [3] that reported published studies by healthcare setting. We selected studies from this review if they directly or indirectly provided evidence on potential drivers of trends from the empirical analyses. We drew on UK studies where possible, and included international evidence where UK evidence was lacking. We also considered the role of relevant regulatory schemes operating within the UK during our study period.

## Results

**Fig. 1:**
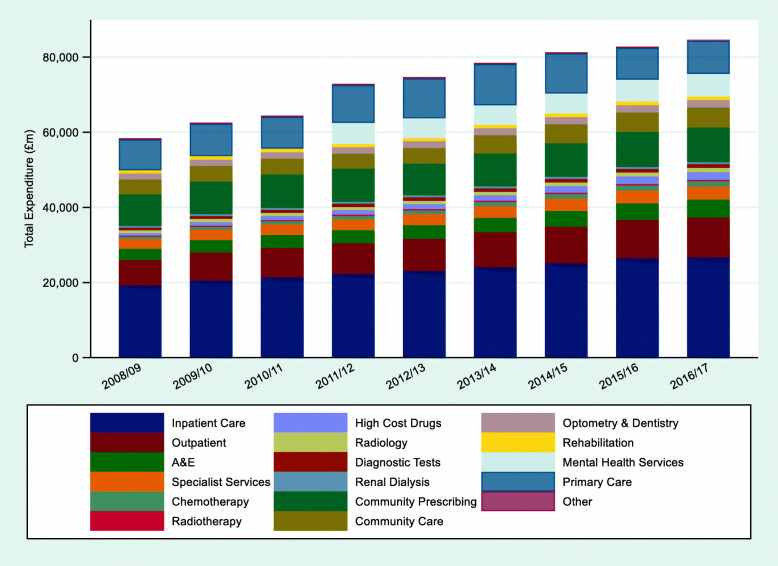
Total expenditure by care setting, £ million

Between 2008/09 and 2016/17, total current expenditure in the English NHS rose from £58.9 billion to £84.6 billion (Fig. 1). NHS expenditure on the three care categories, HBC, D&T and CC, rose by 50.2% and together account for over 82% of NHS expenditure.

For the period 2008/09–2016/17, Table 1 shows the growth in total expenditure, activity and cost and mean year-on-year growth, calculated using Eqs. (3, 1, 2) respectively. The information is provided at setting level as well as weighted averages for the three main groups. Average growth rates are weighted with respect to group size, measured by the relative share of total expenditure for each group. The table also shows the type of activity captured by each of the settings (e.g. FCEs, attendances, items, prescriptions, etc.).

**Fig. 2:**
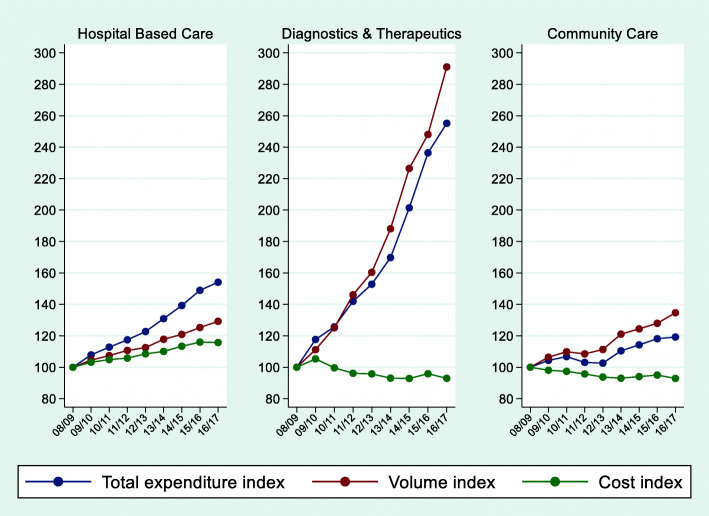
Trends in Expenditure, Activity and Costs: main activity groups

Figure 2 shows the weighted average growth trends for total expenditure, activity and costs for the three broad categories of care HBC, D&T and CC. From 2008/09 to 2016/17, healthcare expenditure and activity rose every year in each of the three groups, with D&T exhibiting the greatest rate of increase. However, the D&T category accounts for approximately 7% of overall NHS spend and so its relative impact is less than that of HBC (which accounts for around 53% of total spend) and also below that of CC (22% of total spend). In terms of cost, there was a positive and increasing trend in HBC for the whole period, whereas the cost trends for D&T and CC were negative. These averages, however, conceal large variations across the different settings, which we consider below.

### Hospital based care (HBC)

Hospital based care (HBC) is the largest expenditure category and includes inpatient, outpatient, A&E and specialist services, accounting for over 50% of total English NHS expenditure. Overall, total expenditure grew by 54.1% from 2008/09 to 2016/17, which corresponds to a 29.2% growth in activity and a 15.7% growth in costs. In other words, around two-thirds of the rise in expenditure was due to increased activity and one-third to rises in cost.

#### Inpatient care

**Fig. 3:**
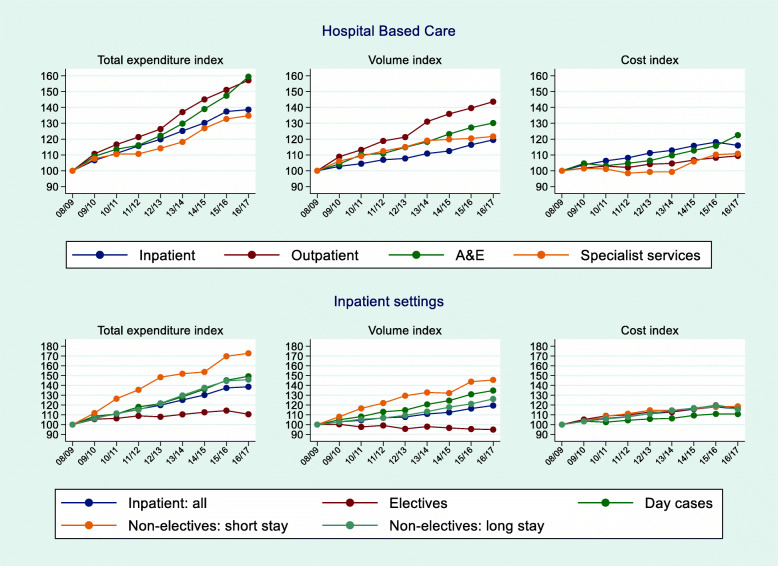
Trends in Expenditure, Activity and Costs for Hospital Based Care and Inpatient care

Figure 3 shows trends for each of the four HBC settings and a further breakdown for inpatient care which is the largest setting in terms of total value, accounting for over one-third of total NHS expenditure. Across the HBC settings, rises in expenditure ranged from 30% to 60% over the nine-year study period.

On average, total inpatient expenditure rose by 4.2% annually. This translates into an increase from 2008/09 to 2016/17 of 38.6% due to rises in both activity (19.5%) and cost (16.0%). There were marked differences in growth rates for elective and non-elective care (Fig. 2). Non-elective inpatient activity grew rapidly (45.6% for short stays and 26.2% for long-stays). In contrast, elective inpatient care fell by 5.1% over the period, whilst day cases rose by 34.8%.

Cost trends for all the inpatient care sub-settings were similar with the rise in total cost ranging from 15% to 19%. The exception was day cases where costs grew just over 10%.

A plausible reason for the switch away from inpatient elective care to day cases is the Best Practice Tariff (BPT). Introduced in 2010, BPTs are national prices designed to incentivise high quality and cost-effective care (‘best practice’) and aim to reduce unexplained variation in clinical quality. The price differential between `best practice’ and `usual’ care creates an incentive for providers to shift from the latter to the former. A notable feature of BPTs is that they incentivise hospitals to admit, treat and discharge patients on the same day (when clinically appropriate) by paying a higher price for day care than for an overnight stay [20]. The fall in inpatient elective care activity (Fig. 3) is more pronounced after 2011/12 and an empirical analysis has confirmed that most BPTs for elective care were effective in achieving this aim [20].

Although demographic factors such as population ageing [21] are associated with rises in inpatient HCE, the ‘red herring’ hypothesis proposes that time-to-death (TTD), rather than age, is the key demographic driver [22] though the interaction of the two factors is also important [23, 24]. However, TTD does not perform well as a predictor of spend on some non-life-threatening conditions such as long-term conditions and diseases treated predominantly with elective inpatient care [25]. It is self-evident that clinical factors, such as morbidities also drive inpatient HCE and indeed, TTD may itself be a proxy for morbidity [26]. A decomposition analysis of English inpatient data showed the prevalence of morbidities had a larger impact on inpatient costs than demographic drivers like age and sex [27]. The interaction between health status and mortality is also important when projecting HCE [28], and relates to the debate on compression and expansion of morbidity [26].

#### Outpatient care

Outpatient figures, which capture care provided by NHS hospital trusts, show that the 57.2% growth in total expenditure was mainly driven by a 47.3% growth in activity whilst the increase in costs was relatively modest (9.4%). These findings are consistent with a Dutch investigation of individual HCE drivers [10], which revealed a move away from inpatient care coupled with a higher rate of day case admissions, shorter inpatient stays and greater use of outpatient clinics. A Spanish study [29] found per capita outpatient expenditure rose by 50% in real terms from 1998 to 2008, with the largest rise in people of working age. Evidence regarding the effect of age and TTD on outpatient utilisation and expenditure is mixed [13, 30]. A US analysis identified that higher use of outpatient care was independently associated with unemployment and also with higher income, suggesting a non-linear relationship between utilisation and socioeconomic status [30]. However, socioeconomic status was not predictive of expenditure at the individual level.

#### Accident & Emergency attendances

The Accident & Emergency (A&E) setting comprises activity performed in Emergency Departments and other A&E services (e.g. ophthalmology, dental, NHS walk in centres). Overall, total expenditure rose by almost 60.0%, translating into a year-on-year rise of 6.0%. This annual rate of increase is at the top of the range cited by a recent systematic review of international studies [31], and in the case of England reflects rises in both activity (30.2%) and cost (22.5%).

An Australian study [32] assessed changes in emergency department visits between 2010 and 2014. The rise in attendance rates per 1000 population exceeded population growth, with the highest rise observed in those aged 85 and over.

The rise in A&E activity could be linked to reduced access to primary care services [31]. There is evidence that A&E is used as an out-of-hours substitute for primary care, and also that younger people perceive A&E as being generally more convenient [31]. Results from the GP (General Practice) Patient Survey for England show that the percentage of people reporting having seen a family doctor in the last 3 months fell by four percentage points between 2011/12 and 2016/17 [33]. A potential explanation is the increasing difficulty in booking an appointment, with the percentage of patients reporting easy access to GP surgery falling by eight percentage points over the same period [34]. These findings suggest that a lack of capacity in primary care could be an underlying reason for the rise in A&E activity. However, the lack of comprehensive data on primary care consultations prevents the computation of growth trends for that setting.

#### Specialist services

In the National Schedule of Reference Costs data, ‘specialist services’ comprises of activity in four distinct services: adult critical care, specialist palliative care, care for cystic fibrosis and – since 2011/12 – cancer multidisciplinary team meetings. Together, these services account for approximately 7.8% of HBC expenditure. Total expenditure rose by 34.8% from 2008/09 to 2016/17 and breakdowns into a growth of 21.7% in activity and of 10.8% in cost.

### Diagnostics & therapeutics (D&T)

**Fig. 4:**
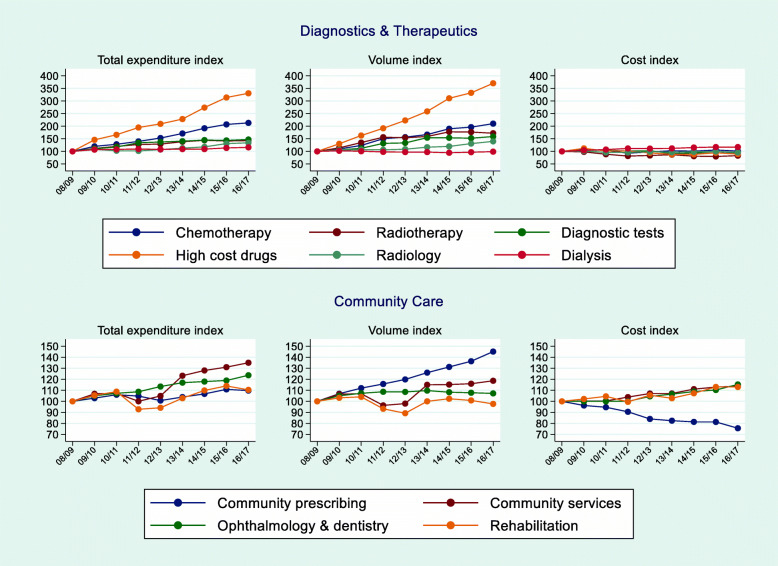
Trends in Expenditure, Activity and Costs for Diagnostics & Therapeutics and Community Care

The Diagnostics and Therapeutics category encompasses six types of care: chemotherapy, radiotherapy, high cost drugs (HCD), radiology, diagnostic tests and renal dialysis. D&T accounted for approximately 7% of total NHS expenditure in England over the study period. Trends for D&T are shown in Fig. 4.

D&T total expenditure grew by 255.2%, driven by an extraordinarily large growth in activity (291.1%) that was slightly offset by a reduction in the cost index (− 7.05%). Activity rose in all types of D&T care, with the exception of renal dialysis (− 1.0%). The largest activity growth was for HCD (270.5%) and chemotherapy (110.2%). Although the patient classification system (Healthcare Resource Groups or HRGs) has been fairly stable since 2013/14, the HRGs used to classify chemotherapy, radiotherapy, and HCD have been subject to substantial revision over time [33]. Better recording of activity and the introduction of new coding that spilt activity in more than one HRG (when previously the activity was captured by a single HRG) could overstate the observed increase in activity.

Nonetheless, the drivers of large rises in activity, and relatively small increases in costs, for HCDs and chemotherapy are worth considering. In England, the availability of new technologies is influenced by appraisals of cost-effectiveness by the National Institute for Health and Care Excellence (NICE) [35]. NICE assesses the value of many HCDs, a category that captures drugs^2^ whose cost is disproportionally high and that are used to treat a limited number of patients. Although NICE assessments inform value-based pricing, NICE does not negotiate the price of new drugs. Over our study period, prices of branded medicines were regulated by a voluntary scheme known as the Pharmaceutical Price Regulation Scheme (PPRS). The aims of the scheme were to keep expenditure on branded medicines within ‘affordable limits’, whilst improving access to new medicines and encouraging innovation [36]. The scheme limited the growth of NHS spend on new drugs, included pricing flexibilities such as Patient Access Schemes (i.e. commercial arrangements), and allowed manufacturers to offer local discounts to hospitals. Therefore, the PPRS is a potential explanation for the observed trends in HCD activity and costs. The Cancer Drug Fund (CDF), which covers the costs of certain drugs that are not recommended by NICE due to their lack of proven cost-effectiveness, was introduced in England in 2011 [37]. The CDF is another plausible driver of the accelerated growth in the volume of HCD observed from 2012/13 onwards.

For settings with negative trends in total cost, values ranged from − 4.1% (radiology) to − 17.4% (radiotherapy). Growth in the cost of chemotherapy was small but positive (1.4%) whereas costs for renal dialysis rose by 17.3%. The reason for the rise in the costs of renal dialysis is unclear, but could be linked to higher levels of multimorbidity [38]. There is also some evidence of positive and linear relationships between TTD and expenditure on D&T [39], which suggests frailty may also be a factor. Other important drivers of HCE are the introduction of new health technologies and institutional characteristics. Evidence from the Netherlands showed that structural factors such as changes in regulation, policy and greater use of new technologies increased costs particularly for the highest cost patients [10].

### Community care (CC)

Community care encompasses community prescribing, community services, optometry, dentistry, and rehabilitation, and accounts for over one-fifth of the total expenditure in the English NHS. Trends for CC are shown in Fig. 4. Overall growth in CC expenditure, activity and cost were 19.2%, 34.7% and − 7.1% respectively, but conceal large variations across settings.

Community prescribing, the largest setting as a share of CC expenditure (55%), exhibits a modest total expenditure growth of 9.8% comprising a 45.2% total activity growth and a fall in cost of 24.4% between 2008/09 and 2016/17. The reduction in pharmaceutical prices may reflect the relatively low price of generics during our study period [40], the Pharmaceutical Price Regulation Scheme [36], and the use of health technology assessment to inform the price of new branded medicines [41, 42]. Our findings contrast with those of a Dutch study [10] which found that prescribing expenditure rose by 69% from 2004 to 2013. The authors found that the increase in expenditure was driven principally by structural shifts such as technological progress (e.g. the highest cost cases were treated with even more expensive drugs). Changes in the distribution of determinants, such as population ageing and a rise in the number of outpatient visits, played a lesser role but were also important explanatory factors. For community prescribing, proximity to death might be a more important driver than age as there is evidence that the effects of age on prescribing expenditure are smaller when models control for TTD [13, 43, 44]. Gender also seems to be a driver of pharmaceutical expenditure: there is evidence that females in all age groups incur higher expenditure [29] and receive more prescriptions [13].

With regard to community services and rehabilitation, activity rose by 35.0% and 10.4% respectively, with steeper rates of increase from 2013/14 onwards. On average, costs rose by around 13% to 14% across the period for both settings. The cost of optometry and dentistry rose by 15%, equating to a mean year-on-year rate of 1.8%, whereas the rise in activity was lower: 7.2% overall, with an average annual rise of 0.9%.

## Discussion

This study of trends in English HCE reveals how much was due to changes in the activity and how much was due to cost, and how this varied across care settings. Overall, HCE grew by approximately 50% over the 9 year study period (2008/09 to 2016/17) driven mainly by a 40% rise in activity, and a comparatively modest growth in costs (7%). Aggregate figures conceal large variations across settings. Specifically, total expenditure on Hospital Based Care (HBC) rose by 54%, spend on Diagnostics and Therapeutics (D&T) rose by 155%, and spend on Community Care (CC) grew by 19%. The rise in HBC expenditure was driven mainly by a rise in activity (29%) but also by a considerable growth in costs (16%).

In the majority of the individual settings, with the exception of renal dialysis and rehabilitation, growth in expenditure was driven primarily by growth in activity. This finding accords with Newhouse’s argument that technological change - “the march of science” – increases the capacity of healthcare systems to supply healthcare [45] and is a major factor driving rising healthcare expenditure. However, whilst there is evidence of a strong, positive relationship between new technologies and aggregate HCE [5, 9], the relationship at the individual level is complex and dynamic, and varies depending on the context and particular type of technology [46]. A better understanding how new technology influences the process of care therefore appears pivotal in determining its impact on HCE and so the financial viability of the future NHS.

HBC is the largest setting within the NHS in terms of overall spend, and also exhibited the largest rise in cost. This points to the need to understand the reasons why cost pressures appear greater in HBC, and future research could examine whether these are due to labour costs, capital costs or factors outside of the HBC setting. Faced with an ageing population and with utilisation rates predicted to continue to increase, greater efficiency may be called for. Alternatively, an improvement in NHS productivity (i.e. the ratio of output growth over input growth) could help alleviate financial pressures. Accounting for 45% of the total input expenditure in 2016/17 [33], labour is the largest single input in the NHS. Therefore, improvements in the labour productivity, such as through reductions in the avoidable use of bank and agency staff, changes in the skill-mix of labour (perhaps via digitally enabled care), or stronger preventative care in ambulatory settings, have potential to curb the growth in HCE.

The NHS Long Term Plan [47] recognises the pressures faced by emergency services. Various remedial measures are proposed, including £4.5 billion new investment in primary care and community care, and the expansion and reform of urgent and emergency care services including the national implementation of ‘urgent treatment centres’ and the roll-out of ‘same day emergency care’ as an alternative to an overnight emergency admission.

Regarding individual drivers, the prevalence of disability, morbidity and multimorbidity appear critical in determining future trends in HCE. International studies have documented changes in the patterns of disability and chronic morbidity, with the age of onset of these conditions occurring later in life (compression of morbidity) [26]. However, the effect on individual lifetime HCE will depend on changes in life-expectancy, and how much of any extra life is disability- or morbidity-free. For example, if individuals live longer and have more years in ill-health (expansion of morbidity) then HCE would likely be higher. Even if morbidity is compressed (fewer years in ill-health), if the complexity of their health needs increases then HCE may also rise. The net impact on aggregate (population level) HCE will also depend on changes in the age structure of the population.

The data used in this study is at an aggregate level. We describe trends in activity, cost and expenditure but can only conjecture how the demand drivers identified in the literature may impact those trends. No causal link is claimed. Moreover, the heterogeneity of the available studies (see [3] for a comprehensive review) makes it very difficult to compare their findings in a robust way. For example, there are large gaps in the evidence for many care settings, and a dearth of studies from the UK. In the future the availability of patient level cost data (PLICS^3^) appears a promising dataset for a more comprehensive study of the HCE drivers at the individual level.

## Conclusions

Our contribution is to shed light on how much each type of setting has contributed to past trends in healthcare expenditure growth and how much of that growth is due to changes in the costs of care or due to changes in the level of activity. Our analyses demonstrate that aggregate trends in HCE mask enormous variation across healthcare settings. This information is useful for policy makers in charge of planning, because it clarifies whether cost pressures or rising activity are the principal reason for rising HCE in the different healthcare settings. Nonetheless, there is a lack of relevant studies for the NHS on how individual drivers affect HCE. Further research is needed to discern the impact of those on cost and to model future healthcare demand.

## Data Availability

The datasets analysed in the current study are freely available to download from the following websites: **National Schedule of Reference Costs (2008/09 to 2016/17).** 2008/09: https://data.gov.uk/dataset/f9b1a80c-187e-4e92-9fe1-25370291f5c0/nhs-reference-costs-2008-09 2009/10–2015/16: https://www.gov.uk/government/collections/nhs-reference-costs 2016/17: https://improvement.nhs.uk/resources/reference-costs/#archive **Prescription Cost Analysis (PCA).** https://digital.nhs.uk/data-and-information/publications/statistical/prescription-cost-analysis **NHS Dental Statistics.** https://digital.nhs.uk/data-and-information/publications/statistical/nhs-dental-statistics **General Ophthalmic Services: activity statistics.** https://digital.nhs.uk/data-and-information/publications/statistical/general-ophthalmic-services-activity-statistics

## Abbreviations

A&E: Accident and emergency (department)
BPT: Best Practice Tariff
CC: Community Care
CDF: Cancer Drug Fund
D&T: Diagnostics and Therapeutics
FCE: Finished Consultant Episode
GDP: Gross Domestic Product
GP: General practice
HBC: Hospital Based Care
HCE: Healthcare expenditure
HRG: Healthcare Resource Group
NHS: National Health Service
NICE: National Institute for Health and Care Excellence
OECD: Organisation for Economic Co-operation and Development
PPRS: Pharmaceutical Price Regulation Scheme
TTD: Time-to-death
US: United States

## Footnotes

1 PCA data are supplied by the Prescription Pricing Authority via the NHS Digital Prescription Drugs Team.

2 The drugs listed vary by year, but include treatments for cancer, hepatitis C, HIV, transplant patients, juvenile arthritis and cystic fibrosis among others.

3 PLICS: Patient Level Information Costing Data Set.
